# Design of Ensemble Stacked Auto-Encoder for Classification of Horse Gaits with MEMS Inertial Sensor Technology

**DOI:** 10.3390/mi9080411

**Published:** 2018-08-17

**Authors:** Jae-Neung Lee, Yeong-Hyeon Byeon, Keun-Chang Kwak

**Affiliations:** Department of Control and Instrumentation Engineering, Chosun University, 375 Seosuk-dong, Gwangju 501-759, Korea; ljn1321@daum.net (J.-N.L.); qasdfghjt@daum.net (Y.-H.B.)

**Keywords:** motion analysis, auto-encoder, dance classification, deep learning, self-coaching, wavelet packet, classification of horse gaits

## Abstract

This paper discusses the classification of horse gaits for self-coaching using an ensemble stacked auto-encoder (ESAE) based on wavelet packets from the motion data of the horse rider. For this purpose, we built an ESAE and used probability values at the end of the softmax classifier. First, we initialized variables such as hidden nodes, weight, and max epoch using the options of the auto-encoder (AE). Second, the ESAE model is trained by feedforward, back propagation, and gradient calculation. Next, the parameters are updated by a gradient descent mechanism as new parameters. Finally, once the error value is satisfied, the algorithm terminates. The experiments were performed to classify horse gaits for self-coaching. We constructed the motion data of a horse rider. For the experiment, an expert horse rider of the national team wore a suit containing 16 inertial sensors based on a wireless network. To improve and quantify the performance of the classification, we used three methods (wavelet packet, statistical value, and ensemble model), as well as cross entropy with mean squared error. The experimental results revealed that the proposed method showed good performance when compared with conventional algorithms such as the support vector machine (SVM).

## 1. Introduction

Riding is an action that includes horse riding or modern equestrian dressage. There are various kinds of horse riding styles, such as show jumping, horse therapy, and so forth. Normally, horse riding requires the skills taught by the coach. However, with the development of technology, motion capture technology has developed and might replace the coach’s role. Motion capture technology is largely divided into acoustical, mechanical, magnetic, and optical sensor. Speaking of their disadvantages, it is difficult for us to collect precise motion using an acoustical sensor, and their movement is restricted because the mechanical type has to wear heavy equipment. Afterwards, optical equipment requires expensive equipment and has a large influence on ambient lighting. Finally, sensors based on magnetic sensors are also sensitive to iron, but horse riding is not closely related to iron. For that reason, magnetic sensors were used.

Normally, horse riding is not taught one-on-one. That is to say, compared with other popularization sports (swimming, badminton, tennis, health), it is very costly. Therefore, horse-riding teaching is acknowledged as an aristocratic sport. For the sake of reducing the education cost of horse riding, a self-coaching system has been designed. In order to do self-coaching system research, the gaits of a horse are classified into different parts: walk, sitting trot, rising trot, and canter. Posture coaching is designed and adjusted according to the gait analysis. In this paper, we do self-coaching research by the means of analyzing horse gait classification. Then, we collect feedback information and show it in the form of voice.

In the past, various designs of the developer were required to extract data characteristics, but deep learning has a positive effect on the developer, because data features can be extracted by themselves. Also, deep learning technology is classified into five main fields of application including natural language processing, customer-centric management, image recognition, and speech recognition [1]. An AE is a type of deep learning method applied in many fields such as video, audio, and text mining. Moreover, an AE is effectively used to develop application services such as motion classification and action recognition. In addition, an AE can extract high-level features from data to train complex features in images [2]. 

There are application categories of an AE that are explained as follows. First, in the field of human motion on accelerometer with MEMS (Microelectromechanical systems), Fourati [3] studied a viable quaternion-based complementary observer (CO) that is designed for rigid body attitude estimation. In particular, the authors used XSENS to analyze human leg, head, and arm movements. Research on the joints of the human body is necessary when performing posture coaching of horse riding. It can constitute dynamic coaching for the movement of a person. Fourati [4] proposed a foot-mounted zero velocity update (ZVU) aided inertial measurement unit (IMU) filtering algorithm for pedestrian tracking in an indoor environment. In particular, the authors developed a coordinate and navigation system to visualize foot motion from 0.12 s to 0.72 s. Although the sensor is attached mainly to the top of the foot, it shows a waveform of a value similar to the data of the sensor attached to the waist of the rider. Since the inertial sensor requires attachment to the body, the user may feel inconvenience. The sensor requires attachment to the body. For that reason, there are a number of studies that have been applied to motion analysis using images through cameras. However, inertia sensors should be used to achieve precise posture analysis [5,6,7,8,9,10,11].

Second, in the field of human motion on deep learning with 2D data, Sarafianos [12] consider the automated recognition of human actions in surveillance videos. Convolution neural network (CNN) is mainly used in 2D, but the author suggested CNN, which is also applicable to 3D. We improved the accuracy of action classification by using frame information in actions. Hasan [13] proposed a continuous activity learning framework for streaming videos by intricately tying together deep hybrid feature models and active learning. Hassan allows us to automatically select the most suitable features and take the advantage of incoming unlabeled instances to improve the existing model incrementally. The authors extracted the characteristics of the encoder through two types of data, not just one. Hong [14] proposed a novel feature extractor with deep learning. It is based on denoising AE and improves traditional methods by adopting locality preserved restriction. The author used data that combines motion data with silhouette data. In previous studies, the AE has been actively studied to fuse two or more data [15,16,17,18,19,20,21,22,23].

In the sports coaching field, Saponara [24] presented a wearable biometric performance measurement system for combat sports, Dhruv [25] researched new integrated technologies that allow coaches, physicians, and trainers to better understand the physical demands of athletes in real time. Extant studies [26] can be a weakness in terms of reliability because of insufficient data. In this study, we constructed a big database, and it is possible to extract a reasonable performance. As data increased, adaptive neuro-fuzzy inference system (ANFIS) [26] faced limitations in performance and time. Therefore, we could solve problems by applying deep learning. Additionally, the ESAE is used for classifying horse gaits. AE can be converted into an ensemble form, which can have a synergistic effect on performance enhancement. In this paper, we propose an ESAE based on motion data of the horse rider. The reason for the classification of horse gaits is that the posture changes according to the horse gaits. The results showed that the angle of each joint was different about gaits of the horse. So, the standard of coaching can be different for each gait. In this paper, the goal is to construct the motion and position database (DB) of the expert rider and compare with the amateur to detect false motion for each horse gaits. This paper also aims to provide real-time horse-riding coaching by providing feedback to the user about posture. The rider’s posture is different for each of the four types of horse gaits. For this reason, the motion of an expert can serve as an example for a beginner. This paper is organized as follows. In Section 2, we describe related research on AEs and SAEs. Algorithms are described in terms of mathematical theorems and concepts. Section 3 describes the proposed method. We describe a feature extraction method using a wavelet packet, and the methods applying five statistical values (maximum value, minimum value, average, variance, and standard deviation) are described. Section 4 describes the DB construction method and the outline of the experiment. In addition, it describes how to build a horse-riding DB. Experiments using the proposed method and comparison algorithm are shown. Section 5 summarizes the conclusions and future challenges.

## 2. Auto-Encoder and Its Variants 

### 2.1. Auto-Encoder

**Figure 1:**
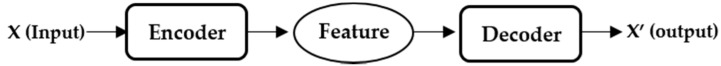
Simple structure of AE.

AE belongs to one of the unsupervised learning algorithms. It is a neural network that aims to produce an output of X’ similar to the input data of X. In other words, AEs are composed of encoding (compression) and decoding (recovery), so data reconstruction is the purpose of AEs. Figure 1 shows the simple structure of the AE. 

**Figure 2:**
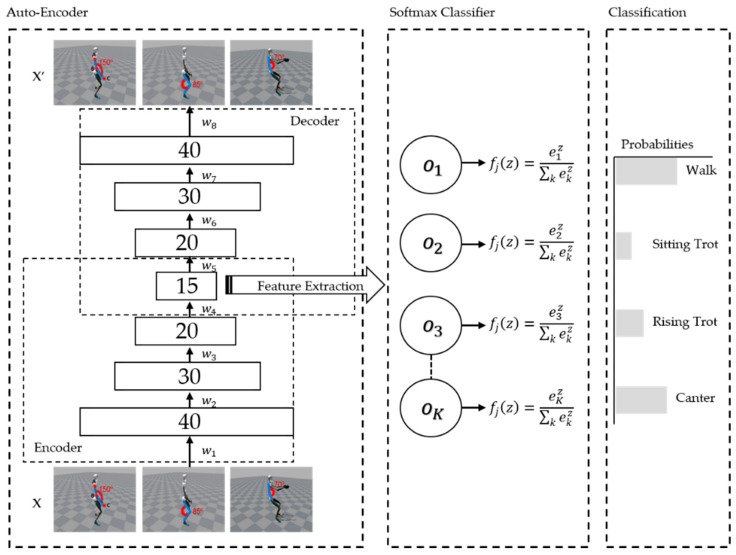
Structure of AE with softmax classifier.

Generally, the encoding process is designed using fewer nodes than the number of nodes in the previous step. For example, if the number of hidden layers of the first AE is set to 40, the number of second AE is set to 30 less than 40. Through the decoding process, the number of hidden layers becomes 40, and the sizes of input data and output data become equal. Figure 2 shows the structure of AE with softmax classifier. Table 1 shows symbols used in defining the learning operation.

AE activates the unit *z* of the hidden layer in the same way as the multi-layer perceptron (weighted sum). *z* is described as Equation (1).

(1)  z= f(x)=σ(Wx+b) 

σ is an active function, mainly a sigmoid function and ReLu function. Next, the auto-encoder decoding step is a result of projecting the new weight value and summing the bias values with *z* obtained in Equation (1). *X*’ is defined as Equation (2).

(2) $$\;X^{\prime }=g\left(y\right)=\;\sigma \prime \left(W\prime z+b\prime \right)\;$$

The difference between the input data *X* and the output data *X*’ is minimized through the objective function *L*.

(3) $$\;L\left(X,X^{\prime }\right)={\left||X-X^{\prime }|\right|}^{2}={\left||X-\sigma \prime (W^{\prime }\left(z\right)+b\prime |\right|}^{2}$$

The objective function *L* is a cross entropy, and it is defined as shown in Equation (4).

(4) $$\;L_{H}\left(X,X\prime \right)=-X_{k}\log X\prime_{x}+\left(1-X_{k}\right)\log\left(1-X\prime_{k}\right]\;$$

Partial differentiation of *L* into weights is shown in Equation (5) using chain rules.

(5) $$\;\frac{\partial L}{\partial y_{i}}\frac{\partial y_{i}}{\partial x_{j}}\frac{\partial x_{i}}{\partial w_{ij}}=-\left(X_{i}-X\prime i\right)x_{j}\;$$

**Figure 3:**
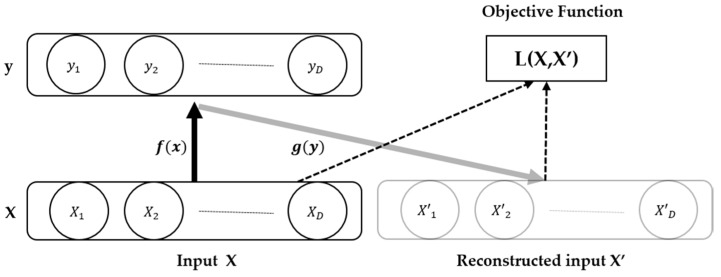
Process of training AE.

Figure 3 shows process of training AE.

Equation (6) represents a weight of output layer of learning operation. The weight of output layer means the connection strength between the hidden layer and the output layer.

(6) $$\;v_{ij}\left(t+1\right)=v_{ij}\left(t\right)+\eta \left(X_{ni}+{x^{\prime }}_{ni}\right)\left(1-{x^{\prime }}_{ni}\right)x_{ni}z_{nj}\;$$

Equation (7) is a learning operation of the hidden layer weight, and the weight of hidden layer means the connection strength between the input and the hidden layer.

(7) $$\;w_{ji}\left(t+1\right)=w_{ji}\left(t\right)+\eta x_{ji}z_{nj}\left(1-z_{nj}\right)\overset{I}{\underset{i}{\sum }}\delta_{ni}v_{ij}\;$$

(8) $$\delta_{ni}=\left(x_{ni}-{x^{\prime }}_{ni}\right)\left(1-{x^{\prime }}_{ni}\right)x\prime_{ni}.\;$$

The bias learning operation of the output layer neuron is shown in Equation (9).

(9) $$\;b_{i}\left(t+1\right)=b_{i}\left(t\right)+\eta (x_{ni}-{x^{\prime }}_{ni})\left(1-{x^{\prime }}_{ni}\right)x\prime_{ni}\;$$

Finally, the bias learning operation of the hidden layer neuron is shown in Equation (10).

(10) $$\;\theta_{j}\left(t+1\right)=\theta_{j}\left(t\right)+\eta \left(1-z_{nj}\right)z_{nj}\overset{I}{\underset{i}{\sum }}\delta_{ni}w_{ij}\;$$

**Pseudocode of Auto-Encoder****Procedure** Auto-EncoderReading:Signal ← horse data← sampling factorTarget signal ← signal samples by KInitialization of variable: Hidden node ← select the number of layers of AEMax epochs ← select the number of layers of AEWeight ← select the number of layers of AESparsity regularization ← select the number of layers of AETraining the AE network (minimum mean square error sense):3.1Feedforward(a)Calculation of outputs at each layer by feeding signal as input to the network.(b)Calculation of error at final layer with reference to target signal3.2Backpropagation of error(a)Calculation of error(b)Back propagating data (error) to previous layers3.3Gradients calculation(a)Calculation of gradients for all weights, biases in all the layers3.4Update(a)Update the parameters using gradient descent mechanism as New parameters ← old parameters—gradientsTesting(a)Test the network using signal against target signal(b)Calculate the error as error ← predicted output—target signalRepeat until convergence     if |error| ≥ tolerance level,       Go to Step.3 else       Finish training


### 2.2. Stacked Auto-Encoder

The stacked auto-encoder (SAE) is a neural network consisting of multiple layers of AE, in which the outputs of each layer are connected to the inputs of the successive layer. SAE requires an enormous amount of computation and risks falling into the local minimum when learning the weights as the number of layers and nodes increases. There is also a problem of vanishing gradient (VG), in which weights are gradually reduced in the process of updating small values continuously. Therefore, a designer can build a network by stacking AEs according to performance. 

The generated network can extract important features from the input data. The parameters of each layer node compare the output and the input data in the output layer through the hidden layer. It can be found that the parameters are determined according to the output data; also, the input data runs in the same way. Once the parameters of a hidden layer are determined, the output layer is removed, and the output of the trained hidden layer is used as input data to design another AE that has a hidden layer and an output layer. An SAE is not a deep generative model. The reason is that RBM depends on probability, and it anticipates test data with input data. However, an SAE trains the model in a deterministic manner. It trains h=s(Wx+b), not p(h=0,1)=s(Wx+b). The advantage of SAE is that the learning speed is fast, and the properties of the deep neural network can be adjusted. SAE stacks block structurally. Primarily, the hidden layer is activated by the input data, and then the active hidden layer reconstructs the input data. 

**Figure 4:**
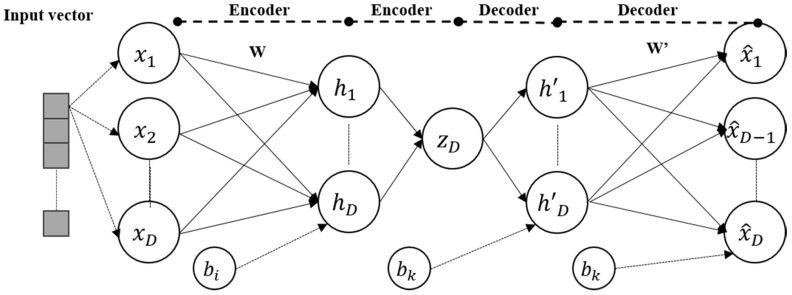
Structure of an SAE.

The error between the input data and the reconstructed data is reduced by using the objective function. The parameters are updated according to activate the hidden layer. In the SAE network, the labels of the sample are also added into the softmax classifier, and the network parameters are tuned using the BP algorithm. Figure 4 shows the structure of an SAE. The target function for fine-tuning the network parameters is as follows:

(11) $$\;J\left(W_{k}{|}_{k-1}^{M},b_{k}{|}_{k-1}^{M}\right)=\mathrm{argmin}\overset{N}{\underset{i=1}{\sum }}{\left||y_{i}-gn\left(fn\left(\ldots f_{1}\left(x_{i}\right)\right)\right)|\right|}_{2}^{2}\;$$

## 3. Proposed Method

### 3.1. Compression Method Using Wavelet Packet

Motion data of a horse rider consists of eight features that are compressed from 49,000 to 12,250 data samples using wavelet packets. The data that pass through the low-frequency filter are extracted as features, and the data that pass through the high-frequency filter are difficult to classify, because they are sparse in terms of their characteristics. High performance was obtained by selecting the two-layer wavelet feature. The computation for the generation of wavelet packets is simple when using an orthogonal wavelet. The sequence of functions shown as follows:

(12) $$\;W_{n}\left(x\right),\;n=0,\;1,\;2,\;\ldots \;$$

**Figure 5:**
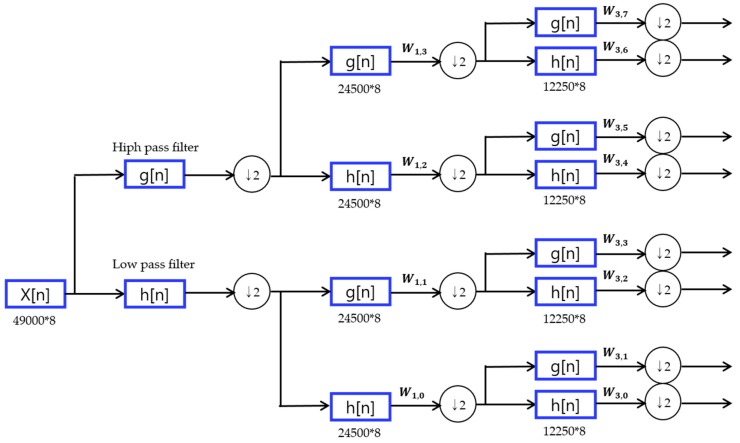
Method of decomposition based on wavelet packet.

We have

(13) $$\;W_{2n}\left(x\right)=\;\sqrt{2}\overset{2N-1}{\underset{k=0}{\sum }}h\left(k\right)W_{n}\left(2x-k\right)\;$$

(14) $$\;W_{2n+1}\left(x\right)=\;\sqrt{2}\overset{2N-1}{\underset{k=0}{\sum }}g\left(k\right)W_{n}\left(2x-k\right)\;$$

in which $W_{0}\left(x\right)=\varphi \left(x\right)$ is the scaling function and $W_{1}\left(x\right)=\varphi \left(x\right)$ is the wavelet function. In this paper, $W_{3,0}$, $W_{2,0},$ and $W_{1,0}$ are used for input. Figure 5 shows the method of decomposition based on wavelet packets.

### 3.2. Feature Extraction Based on Statistical Methods

**Figure 6:**
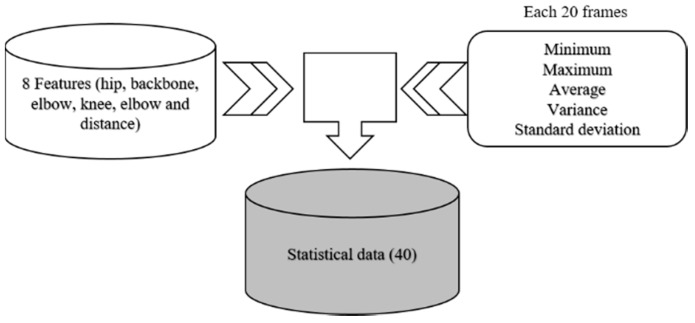
Method of constructing statistical data.

In the previous study [26], we experimented with the elbow angle and the y-axis coordinate data of the hip, whereas in this study, we experimented with forty additional features. Feature values are extracted from 12,000 frames. The feature extraction method gradually extracts five feature values (average, maximum, minimum, variance, and standard deviation) from 1 to 20 frames like mask filter. The data are characterized by eight feature values: y-axis coordinates of the hip, backbone angle, right elbow angle, left elbow angle, right knee angle, left knee angle, elbow distance, and knee distance. We experimented sequentially with 10–100 frames; however, the best performance was achieved at 20 frames, and many features were obtained by taking advantage of the big data feature of AEs. Statistical values are a powerful tool for analyzing time series data. Eight features are generated from the sensor data, and five features are extracted for every frame based on eight features. Finally, 40 features are built. As a result, it is better to apply five feature values than to use a single minimum, a maximum, and an average value. Figure 6 shows a method of constructing statistical data.

### 3.3. Ensemble Stacked Auto-Encoder

The softmax classifier provides us with the probabilities for each class label. It is more convenient for humans to interpret probabilities rather than margin scores of an SVM. The ESAE is constructed by building multiple SAE. By combining the probability values of the classifiers, an ensemble form is created. Thus, we could improve the classification performance by changing the structure. The softmax classifier is defined by Equation (16).

(15) $$\;f_{j}\left(z\right)=\frac{e^{z_{j}}}{\sum_{k}e_{k}^{z}}$$

The ensemble (sum) in the softmax classifier can be denoted as Equation (17). N is the number of SAE.

(16) $$\;\mathrm{Ensemble}\;\left(\mathrm{sum}\right)=\;\frac{\sum_{1}^{N}f_{j}\left(z\right)}{N}\;$$

The ensemble (product) in the softmax classifier can be denoted as Equation (18).

(17) $$\mathrm{Ensemble}\;\left(\mathrm{product}\right)=\;f_{1}\left(z\right)\times f_{2}\left(z\right)\times \;\ldots f_{j}\left(z\right)\;$$

**Figure 7:**
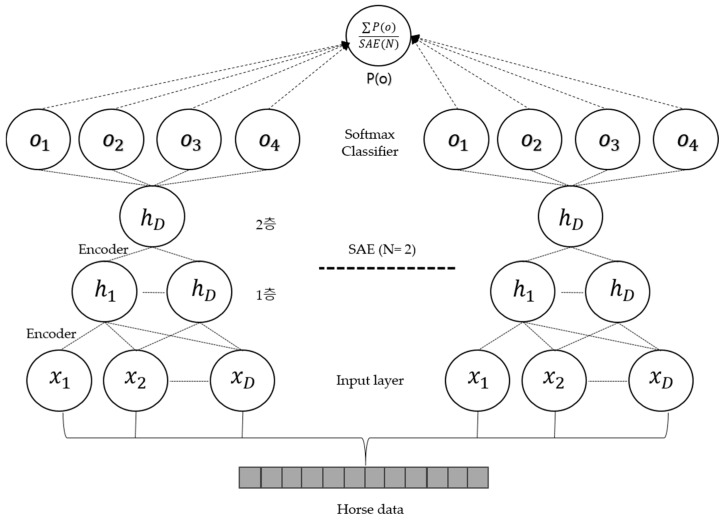
Structure of ESAE.

In ML, ensemble methods use multiple learning algorithms to obtain better predictive performance than what is obtainable from any of the constituent learning algorithms alone. Unlike ML in statistical mechanics, which is usually infinite, an ML ensemble consists of only a concrete finite set of alternative types but typically allows a much more flexible structure to exist among those alternatives. For this purpose, this paper proposes an ESAE. An ESAE consists of two or more SAEs as feature extractors and improves the classification performance by averaging and multiplying the probability values extracted from the softmax classifier using the ensemble (sum) and ensemble (product) values. The performance of the data can be improved owing to the synergy. In this work, we changed the size of hidden nodes in (1) and (2) and experimented in ensemble form. Figure 7 shows the structure of ESAE. The training data is input to SAE (1), SAE (2), and SAE (3) respectively. Learning is performed in the ensemble form according to the change in the hidden layer. Finally, the probability values are obtained from the softmax. Performance is improved through sum and product methods are improved using probability values.


**Pseudocode of Stacked Auto-Encoder**
**Procedure** Ensemble Stacked Auto-EncoderReading:Signal ← horse dataK ← sampling factorN ← number of AETarget signal ← signal samples by KInitialization of variable:Hidden node ← select the number of layers of SAEMax-epochs ← select the number of layers of SAEWeight ← select the number of layers of SAESparsity Regularization ← select the number of layers of SAETraining the SAE network (minimum mean square error sense):(a)Train the AE network of N (2 − N)(b)Obtain probability from softmax classifier(c)Fuse probability values for entire softmax classifier using average and productTesting(a)Test the network using Signal against Target Signal(b)Calculate the error as error ← predicted output—Target SignalRepeat until convergence     if |error| ≥ tolerance level,        Go to Step.3 else Finish training

## 4. Experiment and Database

### 4.1. Sensors of MVN Based Upon Miniature MEMS Inertial Sensor Technology

**Figure 8:**
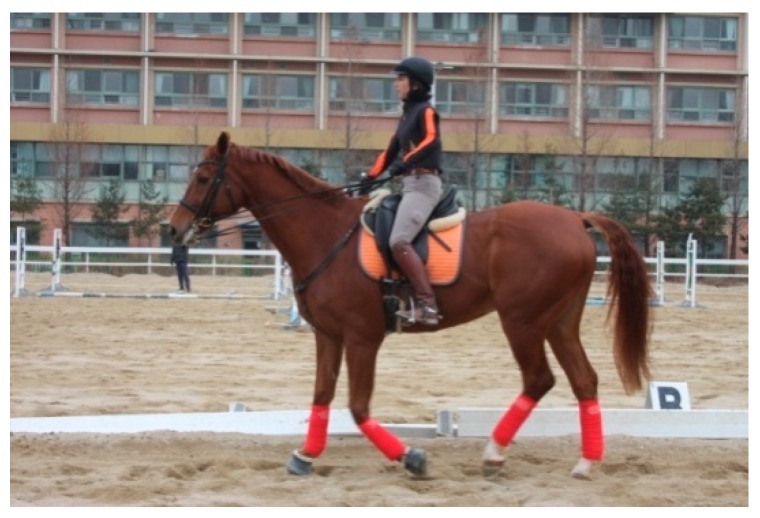
Horse rider with motion capture suit.

Sensors of Xsens are a camera-less 3D human motion measurement system. They are based on state-of-the-art MEMS inertial sensors, biomechanical models, and sensor fusion algorithms. Sensors of Xsens are ambulatory and can be used indoors and outdoors regardless of lighting conditions. The results of the sensor trials require minimal post-processing, as there is no occlusion or lost markers. Results can easily be exported to other software applications. Figure 8 shows a horse rider with a motion capture suit.

### 4.2. Database

Acceleration sensors were used for data acquisition [26]. In order to obtain the reliability of data, data was additionally acquired. Accuracy can be trusted, because data is acquired over many days. There are four types of horse gaits in the database, including walk, sitting trot, rising trot, and canter. The database consists of 40 feature values with 2400 sizes. To describe the 40 feature values, there are eight features such as elbow angles (2), knee angles (2), elbow and knee distance (2), a backbone angle (1), and a hip-y-axis coordinates (1). The 40 features were extracted from 20 frames using mean, maximum, minimum, variance, and standard deviation with eight features. The size of the database for classification is 2400 × 40. The motion data of the horse rider is sized 49,500 × 1. It is projected into each data to obtain the angle data. Owing to the fact that it is an 8-angle value, data of 49,500 × 8 can be constructed. The dimension is reduced through a wavelet packet algorithm. A2 is selected to reduce the dimension, and statistical feature values are extracted through the reduced-size data. 

**Figure 9:**
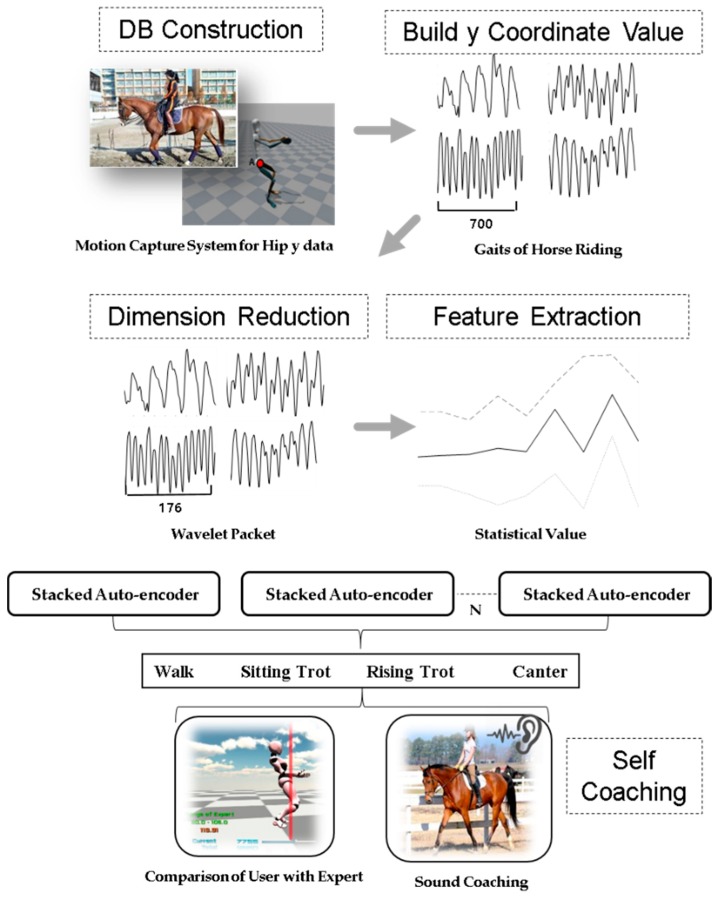
Process of horse-riding coaching.

**Figure 10:**
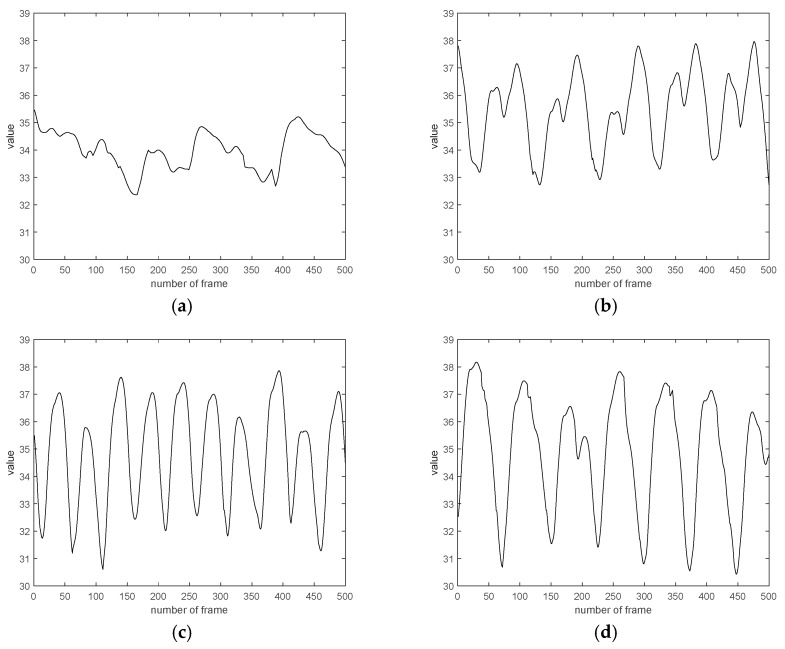
Hip y data with four kind of horse gaits. (**a**) Hip y data for walk, (**b**) hip y data for rising trot, (**c**) hip y data for sitting trot, and (**d**) hip y data for canter.

Several AEs are modeled by using statistical feature values as input to the SAE, and by changing the hidden node. The average is obtained in an ensemble form through the probability values obtained from the respective softmax classifier. When all the results are collected, the final probability value can be obtained. To summarize, there are 4 data. The first data was applied to the AE without preprocessing the data acquired from the sensor to take advantage of the strength of the deep running. Performance is relatively low and excluded. The second data is the hip y data obtained from the sensor with the size 2800 × 70. The third is data using wavelet packet, and the size is 2800 × 18. The parameter settings are as follows. The weights of SAE are set to 0.0001, the max epoch is set to 3000, and the hidden size is 40. Finally, statistical features are extracted after the wavelet packet. The size is 2400 × 40. Training data and testing data is divided 50:50. Figure 9 shows the process of horse-riding coaching. Figure 10 shows hip y data with four kinds of horse gaits [26]. Hip y data with wavelet packet was experimented on in this paper.

### 4.3. Environment

**Figure 11:**
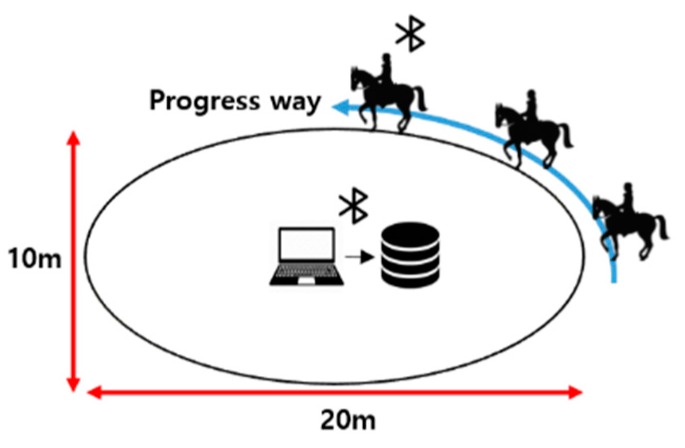
Environment for data acquisition.

Motion data is acquired from an expert of horse riding who made one or two revolutions per gaits (walk, sitting, trot, rising trot, and canter) of an oval horse-riding course 20 m in length and 10 m in breadth while wearing a motion capture suit. Using the 3D motion capture suit based on Xsens inertial sensors, data were extracted in the order of Jeju (137 cm or less), Thoroughbred (160 cm), and Warm Blood (150–173 cm). It took 1 to 2 min to measure a file. Figure 11 shows the environment for data acquisition.

### 4.4. Coaching for Horse Riding

**Figure 12:**
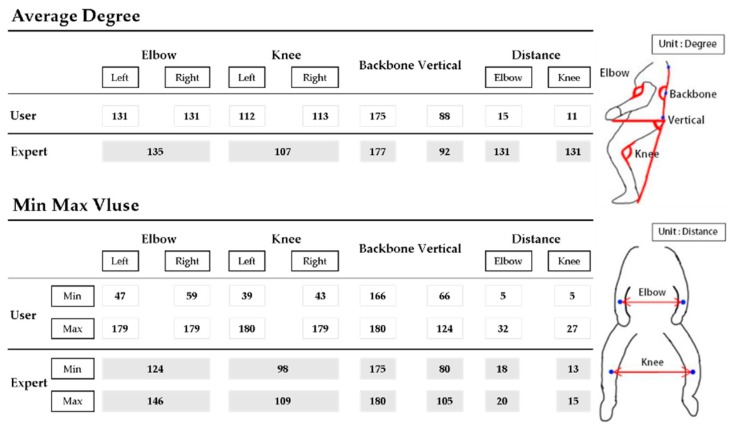
Coaching system for horse riding.

There are various approaches to coaching horse riding, such as muscle utilization, posture, and tacit understanding with a horse. We develop a system that allows users to visualize their data and compare professional posture with amateur posture. Numerically, a range of the maximum value and the minimum value is visually expressed from each feature value. Self-coaching can be achieved by comparing feature values for each frame with an expert. Figure 12 shows a coaching system for horse riding, and Table 2 shows a numerical comparison of walk and canter. 

**Figure 13:**
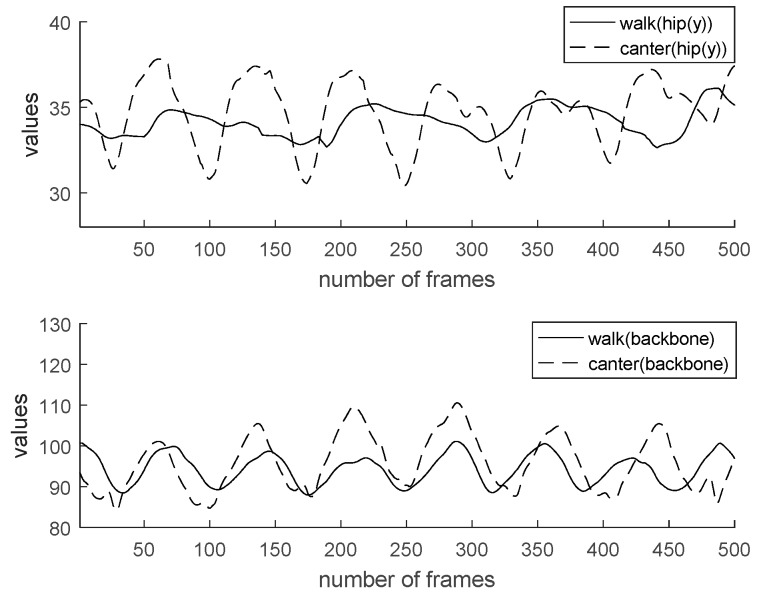
Comparison of two feature values (hip y, angle of backbone) by gaits.

**Figure 14:**
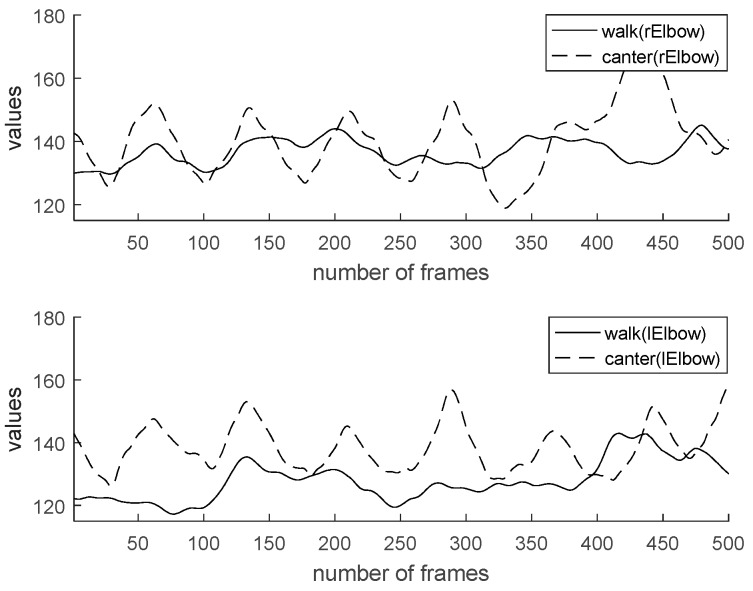
Comparison of two feature values (angle of right elbow, angle of left elbow) by gaits.

**Figure 15:**
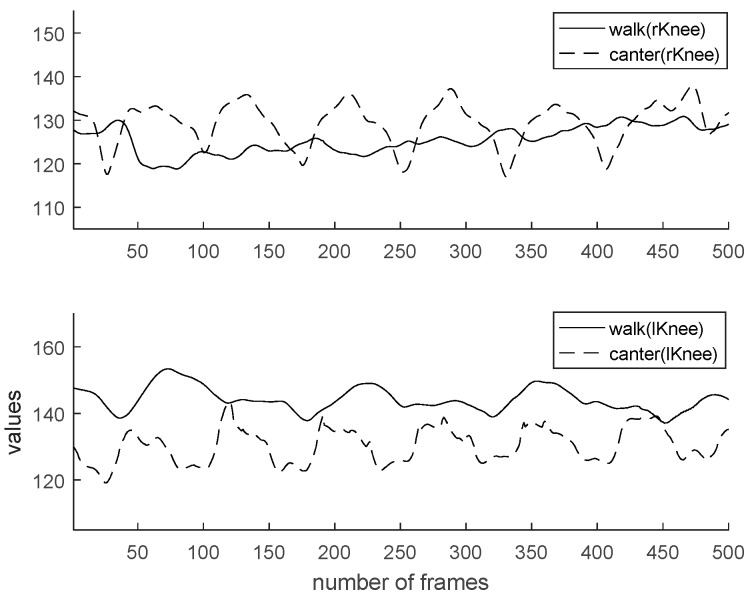
Comparison of two feature values (angle of right knee, angle of left knee) by gaits.

**Figure 16:**
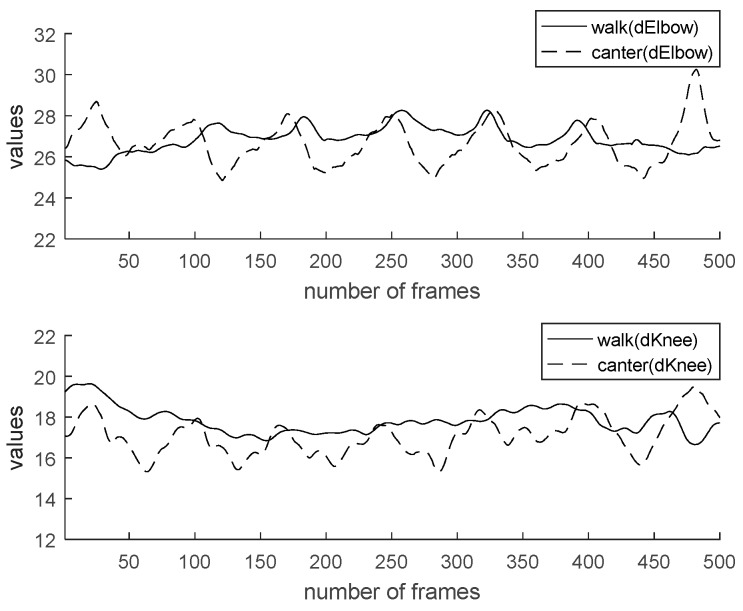
Comparison of two feature values (distance of elbow, distance of knee) by gaits.

Characteristics of walk and canter are analyzed for the sake of coaching. Generally, the data value of horse riding has a cycle. By checking the frame-by-frame period, the user’s motion can be recognized. Figure 13 shows a comparison of two feature values (hip y, an angle of backbone) by gaits. In the case of Figure 13, the change in the value controls the rider’s hip height range. We can see it move more significantly in the canter than in the walk. The canter represents greater movement than the walk regarding elbow angle, knee angle, and backbone angle. Also, the cycle of canter is shorter than walk. Figure 14 shows a comparison of two feature values (an angle of right elbow, an angle of left elbow) by gaits. Figure 15 shows comparison of two feature values (an angle of right knee, an angle of left knee) by gaits. Figure 16 shows a comparison of two feature values (a distance of elbow, a distance of knee) by gaits.

### 4.5. Experiment and Result

**Figure 17:**
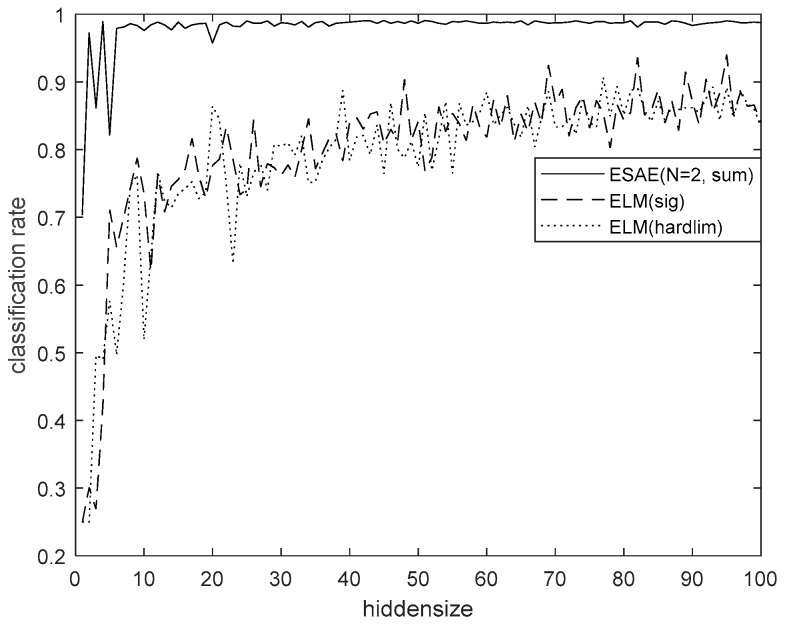
Performance of ESAE for 40 feature data with wavelet packet.

**Figure 18:**
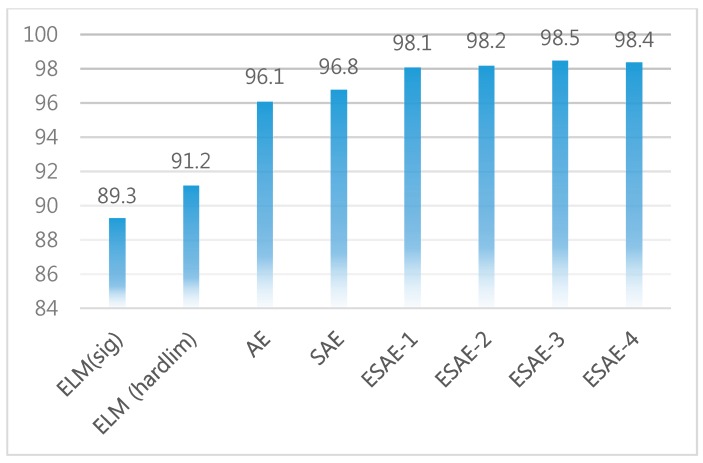
Comparison of performance using 40 feature data with wavelet packet.

This study focused on employing an ESAE to classify horse riding gaits to facilitate real-time coaching. To classify the actual horse-riding gaits, an ESAE with a higher classification rate and real-time posture coaching should be used. In summary, the ESAE exhibited the best performance for classification. According to classification results and the motion information such as the hip value, which is the main parameter for motion analysis and coaching, we can apply the proposed method to the coaching system, for each horse gaits, and for a rider under real or simulated environments. When three SAEs were used, the hidden size of ESAE was set to 30, 20, and 10, respectively, and when two SAEs were used, they were set to 46 and 15, respectively. When a single AE was applied, the average performance was 96.1%, and when a single SAE was applied, the performance was 96.8%. Two kinds of data were used: the hip value and eight characteristic values. The statistical data exhibited good performance in all algorithms, because it could separate all data characteristics well. Among them, the ESAE showed the best performance. Table 3 indicates a comparison of performance using hip y data with wavelet packet. Figure 17 shows the performance of ESAE for 40 feature data with wavelet packet. Figure 18 shows a comparison of performance using 40 feature data with wavelet packet.

### 4.6. Classification Performance

The accuracy Performance of SVM, TREE, KNN, and Ensemble Bagging in Table 3 is used as the ratio of correct classification to the number of total classified samples. The accuracy can be formulized as follows:

(18) $$\;\mathrm{Accuracy}=\frac{\mathrm{TP}+\mathrm{TN}}{\mathrm{TP}+\mathrm{TN}+\mathrm{FP}+\mathrm{FN}}\;$$

TP is the number of correct predictions for positive samples, TN is the number of correct predictions for negative samples, FN is the number of incorrect predictions for positive samples, and FP is the number of incorrect predictions for negative samples. The performance of ESAE models presented in this study is obtained by softmax method described in Section 3.3. 

## 5. Conclusions

This paper proposed the use of an ESAE to classify gaits of horse riding to facilitate real-time coaching. When the ESAE is used, the classification rate is 98.5%. According to classification results, and the motion information such as the hip value, which is the main parameter for motion analysis and coaching, we can apply the proposed method to the coaching system for each horse gait and for a rider under real or simulated environments. Moreover, ANFIS faced limitations in performance and time. Therefore, we could solve this problem by applying deep learning. Additionally, the ESAE is used for classifying horse gaits. AE can be converted into an ensemble form, which can have a synergistic effect on performance enhancement. As future work in the field of horse riding data analysis, we will study aspects of coaching in detail. Further, we plan to employ other body signals in the ESAE algorithm. 

## Figures and Tables

**Table 1:** Symbols used in defining the learning operation.

Symbol	Definition	Symbol	Definition
I	Size of input layer (= output)	J	Size of hidden layer
$\;v_{ij}$	The *j*-th weight of the *i*-th output layer neuron	$\;z_{nj}$	The output value of the *j*-th hidden layer neuron for the *n*-th learning vector
η	Learning rate	$x\prime_{ni}$	The output value of the *i*-th output layer neuron for the *n*-th learning vector
$x_{ni}$	The *i*-th element of the *n*-th learning vector	$\;\theta_{ij}$	Bias of the *j*-th hidden layer neuron
$\;w_{ji}$	The *j*-th weight of the *j*-th hidden layer neuron	$\;b_{i}$	Bias of *i*-th output layer neuron

**Table 2:** Numerical comparison of walk and canter.

Feature	Minimum of Rising Trot	Maximum of Rising Trot	Minimum of Canter	Maximum of Canter
Hip y	32.08	38.79	31.87	38.31
Backbone	171.39	176.47	170.77	176.34
Angle of elbow	127.59	151.82	124.98	159.24
Angle of knee	123.92	172.20	119.50	135.80
Distance of elbow	23.02	27.02	25.87	25.78
Distance of knee	14.93	16.42	15.52	18.59

**Table 3:** Comparison of performance using hip y data with wavelet packet.

Method	Performance
LDA	87.0
SVM	94.5
TREE	84.1
KNN	94.0
Ensemble Bagging	91.3
ELM (Sin)	91.8
ELM (Sig)	91.1
AE (Hidden Size = 15)	94.1
SAE (Hidden Size = 10, 15)	94.2
ESAE-1 (*n* = 3, sum)	94.2
ESAE-2 (*n* = 3, product)	94.3
ESAE-3 (*n* = 2, sum)	95.3
ESAE-4 (*n* = 2, product)	95.2
